# Feeding Broilers with Reduced Dietary Crude Protein or Reduced Soybean Meal Diets Has No Negative Impact on the Performance and Carcass Traits While Reducing the Feed Global Warming Potential †

**DOI:** 10.3390/ani15121753

**Published:** 2025-06-13

**Authors:** Bart Matton, Adriaan Verhelle, Lise Vlerick, Katrijn Keirsschieter, Behnam Saremi

**Affiliations:** 1 CJ Europe GmbH, Unterschweinstiege 2-14, 60549 Frankfurt am Main, Germany; b.matton@cj.net; 2 Poulpharm BV, Prins-Albertlaan 112, 8870 Izegem, Belgium; adriaan.verhelle@poulpharm.com (A.V.); lise.vlerick@poulpharm.com (L.V.); katrijn.keirsschieter@poulpharm.com (K.K.)

**Keywords:** broiler, crude protein, soybean meal, amino acids, nutrition, global warming potential

## Abstract

One way to reduce the environmental impact of the European poultry industry is to feed birds with low crude protein (CP) or low soybean meal (SBM) diets, leading to less SBM import. In this paper, the objective was to examine if low CP and a feed limitation of SBM could be applied to reduce the global warming potential (GWP) of feed without a negative impact of the performance and slaughter parameters. Male Ross 308 birds (n = 1350) were divided between six treatments in a completely randomized design. In a three-phase feeding system, the dietary CP was reduced either only in the finisher phase (from 19% to 17% CP), in the grower phase (from 20% to 19% CP) and finisher phase, or in the starter (from 21% to 20% CP), grower, and finisher phases. In two additional groups, SBM inclusion in feed was reduced in each life phase either to a maximum of 15% SBM (low) or 0% SBM (zero) compared to a positive control (PC) group based on Aviagen 2019 recommendations, modified to 112% for standardized ileal digestibility (SID) arginine (Arg) to lysine (Lys) and 38% for SID histidine (His) to Lys. Data were analyzed with R (Version 4.2.0) using linear regression models. Opteinics™ (Chemovator, Mannheim, Germany) was used to calculate feed GWP. Means were compared with multiple comparisons corrected with Tukey’s test. Low CP diets had no negative effect on performance, carcass weight, and breast meat weight compared to the PC irrespective of the phase in which CP reduction was initiated. Both zero and low SBM groups had superior body weight (*p* < 0.05), weight gain (*p* < 0.05), and FCR (*p* < 0.05) compared to PC at d35. The low and zero SBM groups had higher carcass weight (*p* < 0.05) and breast meat weight (*p* < 0.05) compared to the PC. Both low CP and limiting SBM inclusion in feed positively improved the feed GWP compared to the PC. In conclusion, low CP diets can be applied in broilers during a 35 d growth period or specific phases thereof, with no negative impacts on performance parameters. Low and zero SBM-based diets showed superior performance and carcass weight in comparison to a conventional SBM-driven diet. Both strategies can be used to reduce the GWP of feed.

## Simple Summary

Dietary protein reduction is an important tool to reduce the livestock farming emissions. Soybean meal reduction in broilers’ feed and its substitution with locally sourced raw materials rich in protein is an alternative way to reduce the environmental impact. To combine both methods without a loss in performance depends on a lot of factors such as meeting amino acid requirement properly. In this paper, first, a reduction in the dietary crude protein content in an age-dependent manner was investigated keeping amino acids ratios fixed. Second, the dietary protein content was kept fixed, while soybean meal inclusion in feed was maximized to 15% and to 0% in all growth phases. Soybean meal was replaced with locally sourced protein-rich raw materials fortified with supplementary amino acids to meet the birds’ requirements. The birds fed with low crude protein diets performed similar to the birds fed with a normal crude protein diet. However, limiting soybean in feed to a max of 15% and 0% improved the performance of the birds compared to the birds without a limitation in soybean inclusion. This research indicates that reducing dietary crude protein and reducing soybean meal has a positive impact on the feed global warming potential while maintaining the performance of broilers.

## 1. Introduction

Feed is the major contributor to greenhouse gas emissions in broiler and swine production systems [1]. Reducing crude protein (CP) concentration in diets combined with the use of feed-grade amino acids (AA) are effective strategies to reduce the environmental impact of livestock production, such as climate change, acidification, and eutrophication [2]. Lowering the feed CP content reduces nitrogen (N) excretion in the form of uric acid from birds, thus lowering their environmental impact [3,4]. On the contrary, a high CP diet has the potential to increase the overall load of undigested protein in the hindgut, which is fermented by the microbiota producing toxic metabolites such as biogenic amines, ammonia, phenols, indoles, cresol, branched chain fatty acids, skatole, and hydrogen sulfide [5]. Amines are toxic to the animals and cause growth depression, while others such as ammonia are also detrimental to the environment besides being toxic to the animals.

Recent studies [6,7,8] applied synthetic amino acids, such as lysine (Lys), methionine (Met), threonine (Thr), valine (Val), arginine (Arg), isoleucine (Ile), tryptophan (Trp), and glycine (Gly), to reduce the dietary CP content up to 3% point (pt). The CP content was reduced to 17.5–17.8% and 16.5–16.8% in the grower and finisher phases, respectively, while the starter phase was stable. In contrast, Aderibigbe et al. [9] successfully reduced the dietary CP content in all growth phases beyond 2% pt without a negative impact on the growth rate and meat yield. However, the authors used non-bound AAs (NBAAs), except L-histidine (His), and did not report the digestible His content of the diets. Different attempts to reduce dietary CP content have led to a variety of outcomes; thus, maintaining essential amino acids (EAAs) to Lys ratios and the first-limiting non-EAAs (NEAAs), such as Gly + Serine (Gly + Ser), are key to avoid a loss in performance [10], in addition to the lack of knowledge about requirements of some (lower-limiting) EAAs. This gap is partly due to a paucity of scientific data and partly due to a lack of guidelines for the common broilers used in the industry [11,12]. A recent meta-analysis on low dietary CP in broilers [10] indicated that only 9.6% of the diets were supplemented with L-His, even though His is an EAA in poultry. Recent research [13,14,15,16,17] has shown that standardized ileal digestible (SID) Arg to Lys ratios proposed by a breeder company (106–112% SID Arg to Lys) [11] might not be sufficient in current genetics. In the current study, we balanced the feeds to meet the birds’ presumed requirements for EAAs. The aim was to obtain 112% for SID Arg to Lys and 38% SID His to Lys in all phases, as both His and Arg are commercially available.

Decreasing the flux of undigested protein is not solely related to the absolute amount of CP supplied (quantity), but also the digestibility of protein (quality) [5]. Soybean meal (SBM) is an interesting protein and AA source. In the European Union (EU), SBM has environmental concerns, especially SBM originating from South America, with land use change issues accompanied by significant greenhouse gas emissions [18]. Other environmental concerns such as water consumption and pollution, land occupation, and social concerns connected to soybean cultivation are reviewed by Jia et al. [19]. As a large importer of SBM, the EU feed industry is under pressure to reduce the environmental impact of feed, thus the quest to reduce imported SBM.

In this paper, in the first section (Sec1), the performance and global warming potential (GWP) of birds fed with a low CP diet (applied in a phase-dependent manner) were compared with those of birds fed with a normal CP diet. In the second section (Sec2), the GWP and performance of birds fed with diets formulated to have a partial reduction (maximum of 15%) or a full elimination of SBM (zero) were compared to those of birds fed with a diet without a maximum SBM.

## 2. Materials and Methods

### 2.1. Ethics

The study was carried out according to the “Directive 2010/63/EU of the European Parliament and of the Council of 22 September 2010 on the protection of animals used for scientific purposes” and according to the EFSA [20].

### 2.2. Animals and Housing

A total of 1350 one-day-old male Ross 308 birds obtained from a commercial hatchery (Vervaeke-Belavi, Tielt, Belgium) were housed in the test facility of Poulpharm BV (Izegem, Belgium). Birds were vaccinated against Infectious Bronchitis (d0, d15), Newcastle Disease (d15), and Infectious Bursal Disease (d7, d21). Upon arrival, birds with normal clinical conditions were divided into six groups (15 pen replicates each) in a three-phase feeding experiment (0–12 d, 13–21 d, and 22–35 d). Each floor pen (1 m^2^) was bedded with wood shavings. The birds had unlimited access to water and feed. From d1 to d7, light was reduced stepwise from 24 h to 18 h light per day. Light was then kept at 18 h light and 6 h dark till the end of the trial (d35). Upon arrival, the room temperature was 36 °C. The temperature was gradually reduced until d28 to 22 °C. Then, the temperature was kept at the same level till the end of the trial (d35).

### 2.3. Study Design and Diets

In Sec1, the positive control (PC) treatment (T01) contained a dietary CP level of 21%, 20%, and 19% in the starter, grower, and finisher phases, respectively. The dietary CP levels were reduced either only in the finisher phase (T02: from 19% to 17% CP), in the grower and finisher phases (T03: from 20% to 19% CP and from 19% to 17% CP, respectively), or in the starter, grower, and finisher phases (T04: from 21% to 20% CP, from 20% to 19% CP, and from 19% to 17% CP, respectively) (Table 1).

In Sec2, the inclusion level of SBM was reduced either to a maximum of 15% SBM (low) (T05) or 0% (zero) SBM (T06) compared to T01 containing 25–30% SBM (PC), while maintaining the dietary CP levels similar to T01 (PC). Alternative protein sources like rapeseed meal, peas, field beans, and corn gluten meal were used to replace SBM (Table 2, Table 3 and Table 4 and Supplementary Tables S1–S3). In both sections, NBAAs ensured all the diets met the broiler breeder recommendations on AA [21], except for Arg and His, for which we aimed to obtain 112% for SID Arg to Lys and 38% SID His to Lys in all phases. Raw materials were analyzed prior to formulating the diets for AA, moisture, CP, crude fat, crude fiber, and ash by SGS (Hamburg, Germany). In order to keep the practicality of the trial in a European context, neither Gly was added, as Gly has no registration to be used as an AA in animal feed based on [22], nor was the dietary electrolytic balance (dEB) level corrected between treatments (Table 2, Table 3 and Table 4 and Supplementary Tables S1–S3).

### 2.4. Evaluation Parameters

The body weight (BW) and feed intake (FI) were determined at the pen level on d0, d13, d22, and d35 of age. The mortality-corrected average daily gain (MC-ADG), mortality-corrected average daily feed intake (MC-ADFI), and mortality-corrected feed conversion ratio (MC-FCR) were calculated per pen for the following periods (0–13 d; 14–22 d; and 23–35 d). The MC-ADG, MC-ADFI, and MC-FCR were calculated using the following formulas (shown for the period of 0–13 d of age).

$$MC-ADG=\frac{\left(Weight\;pen\;d13-Weight\;pen\;d0+Weight\;of\;culled\;or\;dead\;birds\;in\;this\;period\right)}{Number\;of\;birds\;at\;d13*13d\;study\;period+Total\;days\;of\;birds\;culled\;or\;dead}$$

$$MC-ADFI=\frac{\left(FEED\;IN-FEED\;OUT\right)}{Total\;days\;of\;birds\;alive\;at\;the\;end\;of\;the\;period+Total\;days\;of\;birds\;culled/dead}$$

$$MC-FCR=\frac{MC-ADFI}{MC-ADG}$$

For the 2500 g body weight-corrected FCR (FCR2500) and the European production efficiency factor (EPEF), the following formulas were used:

$$FCR2500=FCR\;d0\;to\;d35-\frac{Average\;slaughter\;weight\;at\;d35-2500}{50/100}$$

$$EPEF2500=\frac{ADG\;day0\;to\;d35*\%survival\;rate}{FCR2500*10}$$

The footpad lesions were scored in 4 birds per pen at d22 and d35, according to the Bristol scoring system [23]. The score represents an increase in the severity of pododermatitis and ranges from 0 (no evidence of pododermatitis) to 4 (severe pododermatitis). Four birds per pen were sacrificed at d36 to determine slaughter parameters, i.e., individual live weight, carcass weight, breast weight, and leg weight. Breast and leg yields were calculated based on the weight on d36 and carcass weight according to following formulas.

$$Breast\;meat\;yield\left(\%BW\right)=\frac{Breastmeat\;weight}{Body\;weight\;(d36)}*100$$

$$Breast\;meat\;yield\left(\%carcass\right)=\frac{Breast\;meat\;weight}{Carcass\;weight\;(d36)}*100$$

$$Leg\;yield\left(\%BW\right)=\frac{Leg\;weight}{Body\;weight\;(d36)}*100$$

$$Leg\;yield\left(\%carcass\right)=\frac{Leg\;weight}{Carcass\;weight\;(d36)}*100$$

Global warming potential of feed expressed as kg equivalent of CO_2_ per ton of feed was calculated using Opteinics^TM^ (Chemovator, Mannheim, Germany). Opteinics^TM^ aligns with the global sustainability standards, calculating data based on recognized methodologies. It complies with life cycle assessment (LCA) standards ISO 14040:2006 [24] and ISO 14044:2006 [25] and follows the European Commission’s Product Environmental Footprint (PEF) models. The LCA models adhere to sector-specific guidelines based on PEF and the Livestock Environmental Assessment and Performance Partnership (LEAP) for the livestock sector. Opteinics^TM^ uses the global feed LCA institute (GFLI, Zoetermeer, The Netherlands) as a basis for the raw materials’ LCA data while having its own proximate data for feed additives that have no independently verified LCA data. The feed formulas used in this experiment were uploaded to Opteinics^TM^ and the GWP data were extracted.

### 2.5. Statistics

Data were analyzed with R (Version 4.2.0). The statistical unit was at the pen level for all performance parameters except for slaughter parameters and lesion scores (birds were statistical unit). First, the outliers were removed from the dataset. An outlier was defined as any value lying below or above 1.5 times the interquartile range. Next, mortality was evaluated using a Cox proportional hazard model with treatment as fixed effect. All other performance parameters were analyzed using linear regression models. The treatment group was included as a categorical fixed effect. Post hoc multiple comparisons of all treatments were corrected with Tukey’s method for adjustment. Residual plots were checked to evaluate the model fit. Statistical significance was assessed at *p* ≤ 0.05; *p* > 0.05 and *p* ≤ 0.10 indicate a statistical trend.

## 3. Results

### 3.1. Performance Parameters

Overall, no significant differences in mortality were observed (Table 5). In Sec1, the phase-dependent reduction in dietary CP had no significant effect on the performance of the birds, except for a lower MC-ADFI for T04 compared to T01, T02, and T03 in the starter phase (*p* < 0.05), leading to a lower MC-FCR (*p* < 0.05) (Table 5). During the finisher phase, MC-ADG was increased between T02 and T03 compared to PC (T01) (*p* < 0.05). MC-FCR during this period was lower in T02 (*p* < 0.05) and numerically lower in T03 and T04 compared to the PC (T01). When considering the full trial period, no differences in performance parameters between the low CP diets and the PC were observed. Moreover, there were no differences between the low CP diets (T02, T03, and T04).

In Sec2, the SBM-limited diets had no difference in the CP level compared to the PC (T01). During the starter phase, the 0% SBM group showed a lower MC-ADFI compared to the PC (*p* < 0.05), with similar MC-ADG values, resulting in a better MC-FCR (*p* < 0.05), but not differing from 15% SBM group (Table 5). During the grower phase, the 0% and 15% SBM resulted in a higher MC-ADG and a lower MC-FCR (*p* < 0.05) compared to the PC (Table 5). During the finisher phase, the birds fed with 0% and 15% SBM diets grew faster, causing a higher BW (*p* < 0.05). MC-FCR was improved in the 15% SBM group and the PC (*p* < 0.05), while 0% SBM showed intermediate values, not differing from the PC and the 15% SBM groups (Table 5). The MC-ADFI did not differ in the finisher phase. Over the entire experiment, the 0 and 15% SBM groups showed better MC-ADG and MC-FCR results compared to the PC (*p* < 0.05), with no difference between the low and zero SBM. The feed intake was lower in 0% SBM compared with the PC (*p* < 0.05), while the 15% SBM group was intermediate and only differing numerically. The EPEF2500 value shows a greater value for 0 and 15% SBM compared to the PC (*p* < 0.05).

### 3.2. Carcass Traits

The low CP groups (T02, T03, and T04) showed only numerically different values compared to the PC for carcass weight, while the 15% SBM and 0% SBM groups had higher carcass weights, compared to the PC (*p* < 0.05) (Table 6). Additionally, the same observation can be made for breast weight and leg weight for the reduced CP diets. However, reducing SBM in the diets resulted in a higher breast weight (*p* < 0.05), but no difference in leg weight compared to the PC (Table 6).

When evaluating slaughter yield, a higher breast yield (% of BW and % of carcass weight) was observed in the zero SBM group (T06) compared to the PC (*p* < 0.05). There was no difference in breast yield between the other groups (both as % of BW and carcass). A lower leg yield (% of BW) was observed in the group receiving reduced CP during the grower and finisher phases (T03) and the zero SBM group (T06) compared to PC (*p* < 0.05). In contrast, the leg yield (% of carcass weight) was the highest in the PC group (*p* < 0.05) differing from of all the other groups, except the group receiving a reduced CP regime in all three phases (T04).

### 3.3. Footpad Lesions Scores

Concerning the footpad lesion scores (Table 7), no lesions were observed at d22. At d35, reduced lesions were observed for the groups that received reduced dietary CP only during the finisher phase (T02) and in all three phases (T04) compared to the PC group (*p* < 0.05) (Table 7).

### 3.4. Global Warming Potential

In the starter phase, 1726, 1548, 1283, and 748 kg CO_2_ equivalent per ton of feed were produced in T1–3, T4, T5, and T6, respectively. In the grower phase, 1708, 1541, 1342, and 781 kg CO_2_ equivalent per ton of feed were produced in T1–2, T3–4, T5, and T6, respectively. In the finisher phase, 1612, 1262, 1335, and 810 kg CO_2_ equivalent per ton of feed were produced in T1, T2–4, T5, and T6, respectively.

## 4. Discussion

Reducing CP in broiler diets has several advantages such as improved bird health and welfare, reduced environmental impact (e.g., N excretion and ammonia emission from manure), and improved economics in the form of a reduced feed cost [26]. In general, the ideal amino acid concept, i.e., supplying adequate amounts of EAAs into the diet, should work in most cases when dietary protein is reduced. However, this approach largely depends on the extent to which EAA and NEAA requirements are correctly met. The presumed requirements of some (lower-limiting) EAAs in the ideal amino acid concept are not yet fully implemented in practical diets, despite their commercial availability. This gap is partly due to a paucity of scientific data on requirements and partly due to a lack of guidelines in the breeder manuals of common broilers used in the industry [11,12]. For example, requirement studies of His and phenylalanine (Phe) in birds are scarce and are currently not taken up in the breeders’ manuals for common meat broilers types such as Ross 308 and Cobb 500. The results from a recent meta-analysis on reduced dietary CP in broiler diets indicated that 20.5% and 9.6% of the diets were supplemented with L-Phe and L-His, respectively [10]. A recent study [27] suggested that the optimal range for digestible His to Lys for Cobb 500 broilers between d22 and d42 lies between 35 and 38%. Arg is taken up in the broiler breeder manuals; however, recent research [13,14,15,16,17] has shown that the SID Arg to Lys ratios proposed by the broiler breeder companies (106–112% SID Arg to Lys) [11] might not be sufficient to exploit the maximum potential of current genetics. Recent research has proven that reducing dietary CP without performance loss is possible [26]; however, the study period (life phase) may differ and could have a potential influence on the outcomes. For a summary of the study periods used and outcomes, Woyengo et al. [26] comprehensively reviewed the papers until the year 2020 using at least Lys, Met, Thr, and Val and a selection of NEAAs (dispensable AAs such as Gly + Ser, proline, asparagine, and alanine). Using ±10 d old male Ross 308 birds, it was possible to reduce the dietary CP content from the grower phase with 3% pt [6,7,8]. Using male Ross x Ross 708 birds, Aderibigbe et al. [9] tested a reduced dietary CP content from d1 in a three-phase study (1–14 d; 15–25 d; and 26–33 d) up to 3.4% pt in the starter and grower and 2.3% pt in the finisher phase. All authors used NBAAs to correct EAAs up to Ile, tryptophane (Trp), and His, except van Harn et al. [6] and Aderibigbe et al. [9] who did not use His. In one case [7], besides the correction up to His, supplementary non-bound leucine (Leu) was used, as the tested diets were wheat-based (low in Leu). This contrasts with the works of others [8,9], who used a mainly corn-based diet (high in Leu), and those of others [6] who used a combination of both corn- and wheat-based diets.

In Sec1, the basal dietary CP content in the grower and finisher phases (T01) are about 0.5–0.8% pt below the levels previously reported [6,7,8]. In contrast, our basal CP concentration was 2.23, 0.69, and 0.13% pt lower compared to the basal diets used by Aderibigbe et al. [9] in the starter, grower, and finisher phases, respectively. In the aforementioned trials, the CP level was reduced up to 3% pt (17.5–17.8% and 16.5–16.8% in the grower and finisher phases, respectively). In this trial, we reduced the CP level in the starter and grower periods by 1% pt (to 20 and 19% CP, respectively) and in the finisher one with 2% pt (to 17% CP). Compared to Aderibigbe et al. [9], our final CP levels are 0.19–1.69 and 0.13% pt higher for the starter, grower, and finisher phases, respectively. No difference in end BW was also presented in [6,7,8,9]. Similar to Benahmed et al. [8], no difference in MC-FCR and MC-ADFI was observed compared to the control group, independent from the stage in which the dietary CP reduction was applied. A reduction in FCR was observed when reducing the dietary CP level with 3% pt [6], contrary to the works of others [7], who observed a higher feed intake when reducing the dietary CP level with 3% pt; however, this effect was attenuated when the dEB level was restored to the levels of the basal diet. Additionally, supplementing EAAs up to Arg was reported to allow a 2% pt CP reduction without performance loss. In terms of carcass parameters, no differences in terms of live weight, carcass weight, leg weight, and breast weight and yield (as % of carcass or % of live weight) was previously reported [7,8,9]. However, van Harn et al. [6] observed a reduced breast yield (as % of carcass weight) when reducing dietary CP up to 2 or 3% pt. Leg yield (as % of carcass) was reduced in T02 and T03, and a reduction in leg yield (as % of BW weight) in T03 compared to T01 was observed, contrary to Lambert et al. [7]. They reported a difference in leg yield in up to 3% pt dietary CP reduction; the same observation up to 2% pt dietary CP reduction was made by van Harn et al. [6]. These authors [6] used higher arginine levels (111% to 114% SID Arg to Lys, based on apparent fecal digestibility in the grower and finisher phases, respectively) compared to other studies, keeping a minimum of 105% SID or fecal-digestible Arg to Lys ratio. In the current study, a SID Arg to Lys ratio of about 112% throughout the trial was applied, based on earlier work suggesting that the need for Arg is higher than those of the current recommendations [13,14,15,16,17]. Additionally, the SID His to Lys ratio was kept at a minimum of 38%, while a minimum SID His to Lys ratio of 37% was previously used in one study [8] and a minimum 36% fecal-digestible His to Lys in another study [7] or not reported at all [6,9]. A recent study [27] suggested that the optimal range for the SID His to Lys ratio for Cobb 500 broilers, between 22 days and 42 days, lies between 35% and 38%. The variability in the reported requirement of His in broilers [27,28] might stem from a labile pool of His donors available in the body, such as carnosine, part of the His-containing dipeptides [29]. Indeed, a study comparing fast-growing birds to slow-growing birds suggests that the available His is prioritized for growth rather than for storage as His-containing dipeptides [30]. This might explain why potentially small shortages in dietary His can be compensated by the body reserves. Lastly, the SID Phe + tyrosine (Tyr) to Lys ratio (min 107% in all phases) were kept close to the presumed requirement set at 105% and 112% SID (Phe + Tyr) to Lys [28,31]. The used SID (Phe + Tyr) to Lys ratio is therefore not considered deficient in the current experiment. In general, specific attention was paid to the latest scientific insights in correcting the lower-limiting EAAs to the presumed requirements, which might explain the results obtained in this study.

A lack of N supply to synthesize NEAAs is possible after a reduction in the dietary CP concentration. In broilers, a well-researched NEAA is Gly + Ser, also referred to as Gly equivalent (Gly_eq_), and is calculated as follows: Gly (g/kg) + 0.7143 × Ser (g/kg) [32]. As a first-limiting NEAA in poultry diets, adjusting Gly_eq_ in low CP diets is beneficial in maintaining performance [32,33]. The requirement of Gly_eq_ can depend on its precursors like Thr and choline, and the need to metabolize cysteine from Met. Because Gly_eq_ is needed for uric acid formation, reduced urinary N excretion is the major determinant to the response to Gly_eq_ [33]. Thus, in low CP diets, the need for Gly_eq_ might be reduced due to the reduced N excretion or a more efficient N utilization. A minimum of 140% total Gly + Ser to fecal-digestible Lys ratio [7], 135% SID Gly + Ser to Lys [8], and 150% apparent fecal-digestible Gly + Ser to Lys [6] has been applied in low CP diet experiments. In one study [9], only a minimum total Gly + Ser to SID Lys level of 170% during the starter phase was applied. To keep the current trial practical for the EU industry, the NBAA Gly was not used because it is not allowed to be used as an AA in animal feed [22]; consequently, Gly + Ser levels were not corrected. The total Gly + Ser to SID Lys ratios in this study were above 133%, 145%, and 133% in the starter, grower, and finisher phases, respectively. Previously, Mansilla et al. [34] suggested that a minimum content of Gly + Ser should be considered in early phases; therefore, SID Gly + Ser to Lys ratios in the range of 144–149% in grower I, 142–151% in grower II, and 140–152% in the finisher phase were applied. Considering the equal performance and carcass parameters between the reduced CP treatments and the PC group in this trial, the Gly + Ser levels seems not deficient.

A decrease in the dietary CP content leads to a decrease in the SBM inclusion rate. This is usually accompanied by a reduction in dietary potassium (K), which has an impact on the dEB [10]. dEB levels around 226 and 211 mEq/kg in the grower and finisher phases, respectively, were used in a low CP trial [7]. In one treatment group, the dEB level was not fully corrected (122 mEq/kg in the grower and finisher phases) by reducing the K-level while maintaining the sodium (Na) and chlorine (Cl) content of feed. No differences in body weight at d30, a higher ADFI, a worse FCR, and European production index compared to the low CP (−3% pt) diet with corrected dEB (226 and 211 mEq/kg in the grower and finisher phases) were observed. The water to feed ratio was reduced when the CP level in the diet was 3% lower compared to the control group and the other treatments (−1% pt and −2% pt), and it was further reduced when dEB was reduced (not corrected), with reduced litter moisture and footpad lesions consequently. This discord between reduced performance and improved welfare parameters in relation to dEB balance in low CP (in combination with reduced or no SBM in the diet) will need further research. The levels in other studies [6,8] were at 210 and 181 mEq/kg, and 196 and 185 mEq/kg in the grower and finisher phases, respectively. The dEB levels of the tested diets were not reported in Aderbigbe et al. [9]. In the current study, the dEB level was not corrected, but the differences were limited between the control group and the other treatments in Sec1, i.e., 15 to 32 mEq/kg, hence resulting in 180 mEq/kg dEB level in the starter and grower phases and 155 mEq/kg dEB level in the finisher phase (Supplementary Tables S1–S3). However, based on the outcomes in Sec1, we do not consider the dEB levels in our study limiting performance or carcass characteristics. A linear decrease in wet litter and foot pad dermatitis has been previously reported [6,7] and was confirmed in this study as well. The overall footpad lesions in T02 and T04 were lower compared to the control group (Table 7).

In Sec2, the dietary CP content was not changed between the PC and the limited SBM groups. Unexpectedly, the performance of the low and zero SBM groups was higher than the PC. Already in the starter phase, the zero SBM group showed a lower MC-FCR, which was also visible in the next growth phases, mainly originating from a reduced feed intake in combination with a higher growth rate. Similarly, the low SBM group had a lower FCR over the entire growth period. SBM is considered to have similar or higher AA digestibility coefficients than the alternative protein sources used in this trial [35]. On the one hand, the processing conditions to remove anti-nutritional factors and/or the origin of SBM can play a role in the digestibility of SBM [36,37,38]. In the current study, the digestibility values of SBM might have been overrated, emphasizing the importance of an accurate estimation of the digestibility coefficients of raw materials. This might also be the reason why we observed a better MC-FCR and MC-ADG in T02 compared to T01 during the finisher phase. In that phase, the SBM level decreased close to 15% and was compensated solely with NBAAs, which are assumed to be 100% digestible [39]. The higher inclusion of crystallin AA’s in the low CP or the reduced SBM diets could lead to a disconnection of absorption speed between protein-bound amino acids and NBAAs [40]. However, it remains to be elucidated if there is a specific ratio between protein-bound amino acids and NBAAs to optimize protein retention [41]. Researchers applied all 20 proteinogenic AAs to investigate if there is an upper limit to NBAA inclusion compared to peptide-bound AAs [42]. No effect on performance in broiler birds was found when increasing NBAA from 19 g/kg to 53 g/kg during the trial period (7–22 d of age). However, a complete substitution of the peptide-bound AAs with NBAAs (86 g/kg) decreased ADG and ADFI. In this paper, in the maximum 15% SBM or zero SBM groups, the increase in NBAA ranges between 30 and 85% for the starter, 38 and 116% for the grower, and 36 and 103% for the finisher phase, respectively. The NBAA are assumed to have 100% digestibility [39]. Hence, the supplied feed of the reduced SBM diets might meet the requirements of AAs better than the control group and could explain the higher performance results. A high dEB content is one of the nutritional factors influencing litter quality and foot pad lesions [43]. Lowering SBM (rich in K) normally reduces the K content of the diet, thus lowering dEB, which in combination with increasing Cl from NBAAs (Lys-and His hydrochloride used in this trial), reduces the dEB further. In this paper, the dEB was reduced in the maximum 15% or zero SBM groups with 24 and 72 mEq/kg, 19 and 67 mEq/kg, and 14 and 50 mEq/kg compared to the PC group in the starter, grower, and finisher phases, respectively. Despite the reduction in SBM and dEB, only a numerical decrease happened in the mean footpad lesion scores. Albeit it was not significant, it suggests that replacing SBM with alternative protein sources and NBAA supplementation does not lead to increased food pad lesions.

The feed GWP was reduced by both low CP and low SBM inclusion in feed. The feed GWP was cut to about half when the inclusion of SBM was set to zero percent. Thus, this strategy accompanied with better performance results sets the ground as a useful tool to reduce the GWP of poultry industry.

## 5. Conclusions

In conclusion, reducing dietary crude protein either only in the finisher phase, in both the grower and finisher phases, or in the starter, grower, and finisher phases resulted in equal performance and carcass characteristics with lower food pad lesion scores. Reducing soybean meal to maximum 15% or fully replacing soybean meal with alternative protein sources resulted in significantly higher performance and a variable impact on carcass characteristics. Further research is warranted to study the impact of dietary electrolytic balance and the next limiting amino acids when reducing the dietary crude protein content. Both low crude protein and limiting soybean meal in feed are worthy to be further investigated as influential means of reducing poultry feed global warming potential.

## Figures and Tables

**Table 1:** Overview of the different treatment groups in section 1 (T01–T04) and section 2 (T05–T06).

Treatment	Diet Description	Birds/Replicate	Replicates
T01	Normal CP ^1^ (Starter: 21% CP, Grower: 20% CP, Finisher: 19% CP)	15	15
T02	Low CP (Starter: 21% CP, Grower: 20% CP, Finisher: 17% CP)	15	15
T03	Low CP (Starter: 21% CP, Grower: 19% CP, Finisher: 17% CP)	15	15
T04	Low CP (Starter: 20% CP, Grower: 19% CP, Finisher: 17% CP)	15	15
T05	Max 15% SBM ^2^ (Starter: 21% CP, Grower: 20% CP, Finisher: 19% CP)	15	15
T06	No (zero) SBM (Starter: 21% CP, Grower: 20% CP, Finisher: 19% CP)	15	15

^1^ CP: crude protein, ^2^ SBM: soybean meal. T01: positive control (PC).

**Table 2:** Ingredient and nutrient composition of the starter diet (0–12 days of age).

	T01 (PC)-02-03	T04	T05	T06
**Ingredient composition**				
Corn	45.56	49.93	36.80	38.38
Soybean meal	30.08	25.92	15.00	-
Wheat	15.00	15.00	15.00	15.00
Soybean oil	3.35	2.56	4.07	2.23
Rapeseed meal	-	-	8.00	10.84
Field beans	-	-	7.00	8.00
Peas	-	-	4.61	8.00
Corn gluten meal	-	-	3.40	10.58
Others ^a^	4.54	4.60	4.24	4.31
L-Lysine HCl ^b^ (79%)	0.33	0.47	0.50	0.83
L-Methionine 100 (99%)	0.40	0.43	0.39	0.36
L-Threonine (98%)	0.25	0.30	0.26	0.30
L-Valine (96.5%)	0.14	0.21	0.18	0.24
L-Isoleucine (90%)	0.11	0.18	0.18	0.26
L-Arginine (99%)	0.20	0.32	0.29	0.51
L-Histidine HCl (72%)	0.04	0.07	0.05	0.08
L-Tryptophane (98.5%)	-	0.01	0.03	0.08
**Calculated and analyzed nutrient composition ^c^ (%)**				
AMEn (kcal/kg) ^d^	3025.00	3025.00	3025.00	3025.00
Crude Protein	21.00 (21.10)	20.00 (20.60)	21.00 (21.40)	21.00 (21.20)
Crude Fat	6.44 (6.40)	5.74 (5.80)	7.34 (7.20)	6.15 (6.10)
Crude Ash	7.18 (5.95)	7.04 (5.67)	6.92 (5.68)	6.56 (5.05)
Crude Fiber	3.00 (2.20)	2.91 (2.20)	3.80 (3.50)	3.80 (3.50)
**Calculated dietary SID ^e^ amino acid content and analyzed total amino acids (g/kg) ^f^**				
Lys	1.25 (1.23)	1.25 (1.24)	1.25 (1.27)	1.25 (1.28)
Met	0.66 (0.61)	0.67 (0.66)	0.65 (0.64)	0.64 (0.62)
Met + Cys	0.93 (0.93)	0.93 (0.97)	0.93 (0.98)	0.93 (1.00)
Thr	0.84 (0.92)	0.84 (0.89)	0.84 (0.90)	0.84 (0.89)
Trp	0.21	0.2	0.2	0.2
Arg	1.41 (1.48)	1.41 (1.49)	1.40 (1.44)	1.39 (1.41)
Ile	0.84 (0.92)	0.84 (0.87)	0.84 (0.90)	0.84 (0.90)
Leu	1.48 (1.70)	1.38 (1.63)	1.51 (1.80)	1.74 (1.99)
Val	0.94 (1.05)	0.94 (1.01)	0.94 (1.00)	0.94 (1.00)
His	0.48 (0.49)	0.48 (0.48)	0.48 (0.49)	0.48 (0.44)
Phe + Tyr	1.48 (1.67)	1.36 (1.54)	1.41 (1.60)	1.42 (1.59)

^a^ Contains limestone, choline chloride, monocalcium phosphate, sodium bicarbonate, salt, and broiler premix. ^b^ HCL = hydrochloride. ^c^ Values into brackets refer to the analyzed values. ^d^ Nitrogen-corrected apparent metabolizable energy. ^e^ Standardized ileal digestible. ^f^ Arg = arginine, Cys = cysteine, His = histidine, Ile = isoleucine, Leu = leucine, Lys = lysine, Phe = phenylalanine, Thr = threonine, Trp = tryptophane, Tyr = tyrosine, Val = valine. Total analyzed content in parentheses. PC: positive control.

**Table 3:** Ingredient and nutrient composition of the grower diet (13–21 days of age).

	T01 (PC)-02	T03-04	T05	T06
**Ingredient composition**				
Corn	41.48	45.64	31.61	34.24
Soybean meal	28.67	24.76	15.00	-
Wheat	20.00	20.00	20.00	20.00
Soybean oil	4.65	3.90	5.00	3.61
Rapeseed meal	-	-	4.14	10.59
Field beans	-	-	8.00	8.00
Peas	-	-	8.00	8.00
Corn gluten meal	-	-	3.17	9.79
Others ^a^	4.27	4.33	3.80	3.76
L-Lysine HCl ^b^ (79%)	0.21	0.33	0.34	0.67
L-Methionine 100 (99%)	0.33	0.36	0.34	0.29
L-Threonine (98%)	0.17	0.22	0.20	0.22
L-Valine (96.5%)	0.09	0.15	0.14	0.18
L-Isoleucine (90%)	0.04	0.11	0.10	0.18
L-Arginine (99%)	0.09	0.20	0.13	0.37
L-Histidine HCl (72%)	-	-	0.02	0.04
L-Tryptophane (98.5%)	-	-	0.01	0.06
**Calculated and analyzed nutrient composition ^c^ (%)**				
AMEn (kcal/kg) ^d^	3107.08	3107.07	3107.07	3107.07
Crude Protein	20.00 (20.00)	19.00 (19.90)	20.00 (20.60)	20.00 (20.50)
Crude Fat	7.65 (8.00)	6.98 (7.30)	8.11 (7.20)	7.38 (7.20)
Crude Ash	6.87 (5.75)	6.74 (5.45)	6.33 (4.96)	6.03 (4.65)
Crude Fiber	2.97 (2.30)	2.89 (2.20)	3.67 (3.30)	3.80 (3.40)
**Calculated dietary SID ^e^ amino acid content and analyzed total amino acids (g/kg) ^f^**				
Lys	1.12 (1.16)	1.12 (1.19)	1.12 (1.18)	1.12 (1.14)
Met	0.58 (0.56)	0.60 (0.62)	0.58 (0.61)	0.56 (0.56)
Met + Cys	0.85 (0.89)	0.85 (0.94)	0.85 (0.92)	0.90 (0.93)
Thr	0.75 (0.88)	0.75 (0.84)	0.75 (0.86)	0.75 (0.80)
Trp	0.21	0.19	0.18	0.18
Arg	1.26 (1.44)	1.26 (1.35)	1.25 (1.30)	1.24 (1.28)
Ile	0.75 (0.87)	0.75 (0.87)	0.75 (0.83)	0.75 (0.80)
Leu	1.43 (1.76)	1.34 (1.59)	1.45 (1.62)	1.67 (2.05)
Val	0.87 (1.00)	0.87 (1.01)	0.87 (0.98)	0.87 (0.99)
His	0.43 (0.48)	0.40 (0.46)	0.43 (0.46)	0.43 (0.46)
Phe + Tyr	1.44 (1.69)	1.32 (1.59)	1.37 (1.48)	1.37 (1.58)

^a^ Contains limestone, choline chloride, monocalcium phosphate, sodium bicarbonate, salt, and broiler premix. ^b^ HCL = hydrochloride. ^c^ Values into brackets refer to the analyzed values. ^d^ Nitrogen-corrected apparent metabolizable energy. ^e^ Standardized ileal digestible. ^f^ Arg = arginine, Cys = cysteine, His = histidine, Ile = isoleucine, Leu = leucine, Lys = lysine, Phe = phenylalanine, Thr = threonine, Trp = tryptophane, Tyr = tyrosine, Val = valine. Total analyzed content in parentheses. PC: positive control.

**Table 4:** Ingredient and nutrient composition of the finisher diet (22–35 days of age).

	T01 (PC)	T02-03-04	T05	T06
**Ingredient composition**				
Corn	39.39	47.93	30.76	29.39
Soybean meal	25.86	17.64	15.00	-
Wheat	25.00	25.00	25.00	25.00
Soybean oil	5.00	3.46	5.00	5.00
Rapeseed meal	-	-	1.05	12.39
Field beans	-	-	8.00	8.00
Peas	-	-	8.00	8.00
Corn gluten meal	-	-	2.67	7.37
Others ^a^	3.95	4.15	3.43	3.23
L-Lysine HCl ^b^ (79%)	0.19	0.46	0.29	0.55
L-Methionine 100 (99%)	0.29	0.36	0.32	0.25
L-Threonine (98%)	0.15	0.25	0.18	0.19
L-Valine (96.5%)	0.06	0.19	0.10	0.13
L-Isoleucine (90%)	0.05	0.19	0.10	0.17
L-Arginine (99%)	0.06	0.30	0.08	0.27
L-Histidine HCl (72%)	-	0.06	0.01	0.02
L-Tryptophane (98.5%)	-	0.01	0.01	0.04
**Calculated and analyzed nutrient composition ^c^ (%)**				
AMEn (kcal/kg) ^d^	3154.88	3154.88	3154.88	3154.88
Crude Protein	19.00 (19.60)	17.00 (17.60)	19.00 (19.30)	19.00 (19.40)
Crude Fat	7.95 (7.80)	6.60 (6.10)	8.02 (7.30)	8.50 (8.20)
Crude Ash	6.44 (5.36)	6.22 (5.06)	5.78 (4.82)	5.62 (4.52)
Crude Fiber	2.93 (2.20)	2.75 (2.10)	3.45 (3.10)	3.99 (3.70)
**Calculated dietary SID ^e^ amino acid content and analyzed total amino acids (g/kg) ^f^**				
Lys	1.04 (1.10)	1.04 (1.03)	1.04 (1.07)	1.04 (1.13)
Met	0.54 (0.53)	0.57 (0.54)	0.55 (0.60)	0.51 (0.50)
Met + Cys	0.80 (0.85)	0.80 (0.81)	0.80 (0.90)	0.80 (0.86)
Thr	0.70 (0.81)	0.70 (0.76)	0.70 (0.81)	0.70 (0.79)
Trp	0.20	0.17	0.17	0.17
Arg	1.17 (1.28)	1.17 (1.20)	1.15 (1.21)	1.15 (1.26)
Ile	0.72 (0.84)	0.72 (0.78)	0.72 (0.78)	0.72 (0.80)
Leu	1.35 (1.58)	1.17 (1.38)	1.37 (1.47)	1.50 (1.82)
Val	0.80 (0.94)	0.80 (0.86)	0.80 (0.87)	0.80 (0.92)
His	0.41 (0.47)	0.40 (0.42)	0.40 (0.39)	0.40 (0.44)
Phe + Tyr	1.36 (1.59)	1.11 (1.31)	1.31 (1.40)	1.26 (1.50)

^a^ Contains limestone, choline chloride, monocalcium phosphate, sodium bicarbonate, salt, and broiler premix. ^b^ HCL = hydrochloride. ^c^ Values into brackets refer to the analyzed values. ^d^ Nitrogen-corrected apparent metabolizable energy. ^e^ Standardized ileal digestible. ^f^ Arg = arginine, Cys = cysteine, His = histidine, Ile = isoleucine, Leu = leucine, Lys = lysine, Phe = phenylalanine, Thr = threonine, Trp = tryptophane, Tyr = tyrosine, Val = valine. Total analyzed content in parentheses. PC: positive control.

**Table 5:** Performance parameters of birds fed with a different CP ^1^ concentration and different SBM ^2^ inclusions in feed.

Treatments	T01 (PC)	T02	T03	T04	T05	T06
CP level (S,G,F) ^3^	21%–20%–19%	21%–20%–17%	21%–19%–17%	20%–19%–17%	21%–20%–19%	21%–20%–19%
SBM inclusion					Max 15%	0%
**Starter (d 0–12)**						
BW ^4^ d 0	44.7 (0.14)	44.7 (0.14)	44.7 (0.14)	44.9 (0.14)	44.8 (0.14)	44.8 (0.14)
BW d 13	395.0 (4.42)	391.0 (4.42)	386.0 (4.42)	385.0 (4.42)	400.0 (4.42)	389.0 (4.42)
MC-ADG ^5^ (g/d/bird)	26.8 (0.34)	26.6 (0.34)	26.2 (0.34)	26.1 (0.34)	27.3 (0.34)	26.5 (0.34)
MC-ADFI ^6^ (g/d/bird)	34.1 (0.56) ^c^	34.0 (0.58) ^c^	33.9 (0.56) ^c^	31.1 (0.58) ^ab^	32.2 (0.56) ^bc^	29.8 (0.56) ^a^
MC-FCR ^7^ (g/g)	1.25 (0.02) ^bc^	1.30 (0.02)	1.29 (0.02) ^c^	1.19 (0.02) ^ab^	1.17 (0.02) ^ab^	1.13 (0.02) ^a^
**Grower (d 13–21)**						
BW d 22	964.0 (9.03) ^ab^	945.0 (8.72) ^a^	954.0 (8.72) ^a^	942.0 (8.72) ^a^	1007.0 (8.72) ^c^	991.0 (8.72) ^bc^
MC-ADG (g/d/bird)	62.9 (0.69) ^a^	61.3 (0.64) ^a^	63.2 (0.639) ^a^	62.2 (0.64) ^a^	67.4 (0.64) ^b^	66.8 (0.64) ^b^
MC-ADFI (g/d/bird)	112.3 (2.81) ^c^	117.7 (2.51) ^c^	115.2 (2.60) ^c^	114.0 (2.51) ^c^	99.9 (2.51) ^b^	88.5 (2.60) ^a^
MC-FCR (g/g)	1.82 (0.05) ^b^	1.86 (0.05) ^b^	1.82 (0.05) ^b^	1.84 (0.05) ^b^	1.49 (0.04) ^a^	1.33 (0.05) ^a^
**Finisher (d 22–35)**						
BW d 35	2293.0 (17.0) ^a^	2341.0 (17.0) ^abc^	2342.0 (17.0) ^abc^	2309.0 (18.2) ^ab^	2402.0 (17.0) ^c^	2378.0 (17.0) ^bc^
MC-ADG (g/d/bird)	102.6 (0.839) ^a^	107.4 (0.810) ^b^	107.1 (0.839) ^b^	104.1 (0.870) ^ab^	107.2 (0.81) ^b^	107.3 (0.84) ^b^
MC-ADFI (g/d/bird)	160.6 (1.58)	162.9 (1.58)	163.0 (1.58)	163.1 (1.69)	163.1 (1.58)	164.9 (1.58)
MC-FCR (g/g)	1.56 (0.01) ^b^	1.52 (0.01) ^a^	1.52 (0.01) ^ab^	1.54 (0.01) ^ab^	1.52 (0.01) ^a^	1.54 (0.01) ^ab^
**Total period (d 0–35)**						
MC-ADG (g/d/bird)	63.6 (0.48) ^a^	65.1 (0.48) ^abc^	65.3 (0.48) ^abc^	64.5 (0.52) ^ab^	66.9 (0.50) ^c^	66.5 (0.48) ^bc^
MC-ADFI (g/d/bird)	100.0 (0.95) ^bc^	103.0 (0.92) ^c^	103.3 (0.92) ^c^	100.0 (0.95) ^bc^	97.5 (0.95) ^ab^	94.5 (0.92) ^a^
MC-FCR (g/g)	1.57 (0.02)^b^	1.58 (0.02) ^b^	1.58 (0.02) ^b^	1.57 (0.02) ^b^	1.46 (0.02) ^a^	1.42 (0.02) ^a^
Mortality (%)	4.0 (0.00)	3.6 (0.49)	1.8 (0.60)	4.9 (0.45)	1.3 (0.67)	0.9 (0.78)
FCR2500 ^8^	1.86 (0.04) ^a^	1.86 (0.04) ^a^	1.85 (0.04) ^a^	1.85 (0.04) ^a^	1.74 (0.04) ^a^	1.70 (0.04) ^a^
EPEF2500 ^9^	327.3 (9.46) ^a^	343.1 (9.14) ^ab^	353.5 (9.14) ^ab^	348.5 (9.46) ^ab^	382.0 (9.82) ^bc^	391.9 (9.14) ^c^

^1^ CP: crude protein. ^2^ SBM: soybean meal. ^3^ S: starter, G: grower, F: finisher. ^4^ BW: body weight. ^5^ MC-ADG: mortality-corrected average daily gain. ^6^ MC-ADFI: mortality-corrected average daily feed intake. ^7^ MC-FCR: mortality-corrected feed conversion ratio. ^8^ FCR2500: Feed conversion rater recalculated on 2500 g body weight. ^9^ EPEF2500: European efficiency factor recalculated on 2500 g body weight. Values in parentheses represent the standard error. ^a,b,c^ Values with different superscripts in the same row differ significantly (*p* ≤ 0.05)—Tukey’s test. PC: positive control.

**Table 6:** Carcass parameters of birds fed with different CP ^1^ concentration and different SBM ^2^ inclusions in feed.

Treatments	T01 (PC)	T02	T03	T04	T05	T06
CP level (S,G,F) ^3^	21%–20%–19%	21%–20%–17%	21%–19%–17%	20%–19%–17%	21%–20%–19%	21%–20%–19%
SBM inclusion					Max 15%	0%
**Day 36**						
Live weight (g)	2297.0 (10.70) ^a^	2342.0 (10.70) ^b^	2339.0 (10.70) ^ab^	2338.0 (10.70) ^ab^	2409.0 (10.70) ^c^	2375.0 (10.70) ^bc^
Carcass weight (g)	1663.0 (10.70) ^a^	1694.0 (10.70) ^ab^	1678.0 (10.70) ^ab^	1676.0 (10.80) ^ab^	1741.0 (10.70) ^c^	1709.0 (10.90) ^bc^
Breast weight (g)	389.0 (5.21) ^a^	408.0 (5.30) ^ab^	406.0 (5.21) ^ab^	407.0 (5.21) ^ab^	422.0 (5.21) ^b^	424.0 (5.21) ^b^
Leg weight (g)	638.0 (5.58) ^a^	634.0 (5.63) ^a^	628.0 (5.58) ^a^	633.0 (5.58) ^a^	649.0 (5.58) ^a^	634.0 (5.68) ^a^
Breast meat yield (% of body weight)	16.9 (0.20) ^a^	17.4 (0.199) ^ab^	17.3 (0.20) ^ab^	17.4 (0.20) ^ab^	17.4 (0.201) ^ab^	17.8 (0.20) ^b^
Breast meat yield (% of carcass)	23.4 (0.24) ^a^	24 (0.247) ^ab^	24.2 (0.24) ^ab^	24.2 (0.24) ^ab^	24.1 (0.24) ^ab^	24.8 (0.24) ^b^
Leg yield (% of body weight)	27.8 (0.21) ^b^	27.2 (0.212) ^ab^	26.2 (0.22) ^a^	27.0 (0.21) ^ab^	27.0 (0.21) ^ab^	26.7 (0.21) ^a^
Leg yield (% of carcass)	38.4 (0.21) ^b^	37.6 (0.211) ^a^	37.2 (0.22) ^a^	37.6 (0.21) ^ab^	37.2 (0.21) ^a^	37.1 (0.21) ^a^

^1^ CP: crude protein. ^2^ SBM: soybean meal. ^3^ S: starter, G: grower, F: finisher. Values in parentheses represent the standard error. ^a,b,c^ Values with different superscripts in the same row differ significantly (*p* ≤ 0.05)—Tukey’s test. PC: positive control.

**Table 7:** Footpad lesions on d 22 and d 35 of the birds fed with a different CP ^1^ concentration and different SBM ^2^ inclusions in feed.

Treatment	T01 (PC)	T02	T03	T04	T05	T06
CP level (starter, grower, finisher)	21%–20%–19%	21%–20%–17%	21%–19%–17%	20%–19%–17%	21%–20%–19%	21%–20%–19%
SBM inclusion					Max 15%	0%
Mean footpad lesion ^3^ score (d22)	0	0	0	0	0	0
Mean footpad lesion score (d35)	1.3 ^b^	0.9 ^a^	1.1 ^ab^	0.8 ^a^	1.0 ^ab^	1.1 ^ab^

^1^ CP: crude protein. ^2^ SBM: soybean meal. ^3^ Footpad lesions were scored in 4 birds per pen at d22 and d35, according to the Bristol scoring system [23]. The score represents an increase in the severity of pododermatitis and ranges from 0 (no evidence of pododermatitis) to 4 (severe pododermatitis). ^a,b^ Values with different superscripts in the same row differ significantly (*p* ≤ 0.05)—Tukey’s test. PC: positive control.

## Data Availability

The original contributions presented in this study are included in this article. Further inquiries can be directed to the corresponding author.

## Supplementary Materials

The following supporting information can be downloaded at: https://www.mdpi.com/article/10.3390/ani15121753/s1, Table S1: Complementary nutrient composition of the starter diet (0–12 days of age).; Table S2: Complementary nutrient composition of the starter diet (13–21 days of age); Table S3: Complementary nutrient composition of the starter diet (22–35 days of age).
