# Supplementary material for: Feeding Broilers with Reduced Dietary Crude Protein or Reduced Soybean Meal Diets Has No Negative Impact on the Performance and Carcass Traits While Reducing the Feed Global Warming Potential
Source: Animals (Basel). 2025 Jun 13;15(12):1753. doi: 10.3390/ani15121753 (PMC12189125; doi:10.3390/ani15121753)
Supplement: Supplementary file 1 [file animals-15-01753-s001.zip › animals-3656421-supplementary.pdf]

Table S1. Complementary nutrient composition of the starter diet (0-12 days of age).

	T01 (PC)-02-03	T04	T05	T06
**Calculated and analyzed nutrient composition [a] (%)**				
Moisture	10.94 (10.63)	10.97 (10.47)	10.84 (10.04)	10.97 (9.89)
AMEn (kcal/kg) [b]	3025.00	3025.00	3025.00	3025.00
Crude Protein	21.00 (21.10)	20.00 (20.60)	21.00 (21.40)	21.00 (21.20)
Crude Fat	6.44 (6.40)	5.74 (5.80)	7.34 (7.20)	6.15 (6.10)
Crude Ash	7.18 (5.95)	7.04 (5.67)	6.92 (5.68)	6.56 (5.05)
Crude Fiber	3.00 (2.20)	2.91 (2.20)	3.80 (3.50)	3.80 (3.50)
Starch	40.00	42.70	39.15	42.81
dEB [c] (mEq/kg)	201.88	185.70	178.16	129.69
**Calculated dietary SID [d] amino acid content and analyzed total amino acids (g/kg) [e]**				
Lys	1.25 (1.23)	1.25 (1.24)	1.25 (1.27)	1.25 (1.28)
Met	0.66 (0.61)	0.67 (0.66)	0.65 (0.64)	0.64 (0.62)
Cys	0.27 (0.32)	0.26 (0.31)	0.28 (0.34)	0.29 (0.38)
Met+Cys	0.93 (0.93)	0.93 (0.97)	0.93 (0.98)	0.93 (1.00)
Thr	0.84 (0.92)	0.84 (0.89)	0.84 (0.90)	0.84 (0.89)
Trp	0.21	0.2	0.2	0.2
Arg	1.41 (1.48)	1.41 (1.49)	1.40 (1.44)	1.39 (1.41)
Ile	0.84 (0.92)	0.84 (0.87)	0.84 (0.90)	0.84 (0.90)
Leu	1.48 (1.70)	1.38 (1.63)	1.51 (1.80)	1.74 (1.99)
Val	0.94 (1.05)	0.94 (1.01)	0.94 (1.00)	0.94 (1.00)
His	0.48 (0.49)	0.48 (0.48)	0.48 (0.49)	0.48 (0.44)
Phe	0.88 (0.99)	0.81 (0.90)	0.82 (0.92)	0.79 (0.90)
Tyr	0.60 (0.68)	0.55 (0.64)	0.59 (0.68)	0.63 (0.69)
Phe+Tyr	1.48 (1.67)	1.36 (1.54)	1.41 (1.60)	1.42 (1.59)
Gly	0.74 (0.82)	0.68 (0.76)	0.72 (0.79)	0.64 (0.71)
**Calculated SID amino acid ratios to Lys**				
M+C/Lys	0.74	0.74	0.74	0.74
Thr/Lys	0.67	0.67	0.67	0.67
Trp/Lys	0.17	0.16	0.16	0.16
Arg/Lys	1.13	1.13	1.12	1.12
Ile/Lys	0.67	0.67	0.67	0.67
Leu/Lys	1.18	1.11	1.21	1.40
Val/Lys	0.75	0.75	0.75	0.75
His/Lys	0.38	0.38	0.38	0.38
Phe+Tyr/Lys	1.18	1.08	1.12	1.13
Gly + Ser Total/Lys SID	1.45	1.33	1.41	1.32

[a] Values into brackets refer to the analyzed values. [b] Nitrogen-corrected apparent metabolizable energy. [c] dEB= dietary electrolytic balance. [d] Standardized ileal digestible. [e] Arg = arginine, Cys = cysteine, Gly = glycine, His = histidine, Ile = isoleucine, Leu = leucine, Lys = lysine, Phe = phenylalanine, Thr = threonine, Trp = tryptophane, Tyr = tyrosine, Val = valine. Total analyzed content in parentheses. PC: positive control.

Table S2. Complementary nutrient composition of the starter diet (13-21 days of age).

	T01 (PC)-02	T03-04	T05	T06
**Calculated and analyzed nutrient composition [a] (%)**				
Moisture	10.87 (10.80)	10.91 (10.89)	10.84 (10.75)	10.94 (10.26)
AMEn (kcal/kg) [b]	3107.08	3107.07	3107.07	3107.07
Crude Protein	20.00 (20.00)	19.00 (19.90)	20.00 (20.60)	20.00 (20.50)
Crude Fat	7.65 (8.00)	6.98 (7.30)	8.11 (7.20)	7.38 (7.20)
Crude Ash	6.87 (5.75)	6.74 (5.45)	6.33 (4.96)	6.03 (4.65)
Crude Fiber	2.97 (2.30)	2.89 (2.20)	3.67 (3.30)	3.80 (3.40)
Starch	40.35	42.93	40.72	43.03
dEB [c] (mEq/kg)	196.98	181.79	178.12	130.47
**Calculated dietary SID[d] amino acid content and analyzed total amino acids (g/kg) [e]**				
Lys	1.12 (1.16)	1.12 (1.19)	1.12 (1.18)	1.12 (1.14)
Met	0.58 (0.56)	0.60 (0.62)	0.58 (0.61)	0.56 (0.56)
Cys	0.27 (0.33)	0.26 (0.32)	0.27 (0.31)	0.29 (0.37)
Met+Cys	0.85 (0.89)	0.85 (0.94)	0.85 (0.92)	0.90 (0.93)
Thr	0.75 (0.88)	0.75 (0.84)	0.75 (0.86)	0.75 (0.80)
Trp	0.21	0.19	0.18	0.18
Arg	1.26 (1.44)	1.26 (1.35)	1.25 (1.30)	1.24 (1.28)
Ile	0.75 (0.87)	0.75 (0.87)	0.75 (0.83)	0.75 (0.80)
Leu	1.43 (1.76)	1.34 (1.59)	1.45 (1.62)	1.67 (2.05)
Val	0.87 (1.00)	0.87 (1.01)	0.87 (0.98)	0.87 (0.99)
His	0.43 (0.48)	0.40 (0.46)	0.43 (0.46)	0.43 (0.46)
Phe	0.86 (0.98)	0.79 (0.92)	0.80 (0.87)	0.77 (0.89)
Tyr	0.58 (0.71)	0.53 (0.67)	0.57 (0.61)	0.60 (0.69)
Phe+Tyr	1.44 (1.69)	1.32 (1.59)	1.37 (1.48)	1.37 (1.58)
Gly	0.72 (0.80)	0.66 (0.73)	0.70 (0.77)	0.64 (0.71)
**Calculated SID amino acid ratios to Lys**				
M+C/Lys	0.76	0.76	0.76	0.76
Thr/Lys	0.67	0.67	0.67	0.67
Trp/Lys	0.19	0.17	0.16	0.16
Arg/Lys	1.12	1.12	1.11	1.11
Ile/Lys	0.67	0.67	0.67	0.67
Leu/Lys	1.28	1.20	1.30	1.49
Val/Lys	0.78	0.78	0.78	0.78
His/Lys	0.38	0.36	0.38	0.38
Phe+Tyr/Lys	1.28	1.18	1.23	1.23
Gly + Ser Total/Lys SID	1.58	1.45	1.54	1.45

[a] Values into brackets refer to analyzed values. [b] Nitrogen-corrected apparent metabolizable energy. [c] dEB= dietary electrolytic balance. [d] Standardized ileal digestible. [e] Arg = arginine, Cys = cysteine, Gly = glycine, His = histidine, Ile = isoleucine, Leu = leucine, Lys = lysine, Phe = phenylalanine, Thr = threonine, Trp = tryptophane, Tyr = tyrosine, Val = valine. Total analyzed content in parentheses. PC: positive control.

Table S3. Complementary nutrient composition of the starter diet (22-35 days of age).

	T01 (PC)	T02-03-04	T05	T06
**Calculated and analyzed nutrient composition [a] (%)**				
Moisture	10.88 (11.10)	10.93 (10.97)	10.90 (10.89)	10.87 (10.40)
AMEn (kcal/kg) [b]	3154.88	3154.88	3154.88	3154.88
Crude Protein	19.00 (19.60)	17.00 (17.60)	19.00 (19.30)	19.00 (19.40)
Crude Fat	7.95 (7.80)	6.60 (6.10)	8.02 (7.30)	8.50 (8.20)
Crude Ash	6.44 (5.36)	6.22 (5.06)	5.78 (4.82)	5.62 (4.52)
Crude Fiber	2.93 (2.20)	2.75 (2.10)	3.45 (3.10)	3.99 (3.70)
Starch	41.96	47.25	43.15	42.53
dEB [c] (meq/kg)	187.02	154.97	172.82	137.00
**Calculated dietary SID [d] amino acid content and analyzed total amino acids (g/kg) [e]**				
Lys	1.04 (1.10)	1.04 (1.03)	1.04 (1.07)	1.04 (1.13)
Met	0.54 (0.53)	0.57 (0.54)	0.55 (0.60)	0.51 (0.50)
Cys	0.26 (0.32)	0.23 (0.27)	0.25 (0.30)	0.29 (0.36)
Met+Cys	0.80 (0.85)	0.80 (0.81)	0.80 (0.90)	0.80 (0.86)
Thr	0.70 (0.81)	0.70 (0.76)	0.70 (0.81)	0.70 (0.79)
Trp	0.20	0.17	0.17	0.17
Arg	1.17 (1.28)	1.17 (1.20)	1.15 (1.21)	1.15 (1.26)
Ile	0.72 (0.84)	0.72 (0.78)	0.72 (0.78)	0.72 (0.80)
Leu	1.35 (1.58)	1.17 (1.38)	1.37 (1.47)	1.50 (1.82)
Val	0.80 (0.94)	0.80 (0.86)	0.80 (0.87)	0.80 (0.92)
His	0.41 (0.47)	0.40 (0.42)	0.40 (0.39)	0.40 (0.44)
Phe	0.82 (0.93)	0.67 (0.76)	0.77 (0.82)	0.72 (0.83)
Tyr	0.54 (0.66)	0.44 (0.55)	0.54 (0.58)	0.54 (0.67)
Phe+Tyr	1.36 (1.59)	1.11 (1.31)	1.31 (1.40)	1.26 (1.50)
Gly	0.69 (0.78)	0.56 (0.64)	0.66 (0.68)	0.64 (0.73)
**Calculated SID amino acid ratios to Lys**				
M+C/Lys	0.77	0.77	0.77	0.77
Thr/Lys	0.67	0.67	0.67	0.67
Trp/Lys	0.19	0.16	0.16	0.16
Arg/Lys	1.12	1.12	1.11	1.11
Ile/Lys	0.69	0.69	0.69	0.69
Leu/Lys	1.3	1.12	1.32	1.44
Val/Lys	0.77	0.77	0.77	0.77
His/Lys	0.39	0.38	0.38	0.38
Phe+Tyr/Lys	1.3	1.07	1.26	1.22
Gly + Ser Total/Lys SID	1.62	1.33	1.58	1.52

[a] Values into brackets refer to analyzed values. [b] Nitrogen-corrected apparent metabolizable energy. [c] dEB= dietary electrolytic balance. [d] Standardized ileal digestible. [e] Arg = arginine, Cys = cysteine, Gly = glycine, His = histidine, Ile = isoleucine, Leu = leucine, Lys = lysine, Phe = phenylalanine, Thr = threonine, Trp = tryptophane, Tyr = tyrosine, Val = valine. Total analyzed content in parentheses. PC: positive control.
